# Genomic Copy Number Variants: Evidence for Association with Antibody Response to Anthrax Vaccine Adsorbed

**DOI:** 10.1371/journal.pone.0064813

**Published:** 2013-05-31

**Authors:** Michael I. Falola, Howard W. Wiener, Nathan E. Wineinger, Gary R. Cutter, Robert P. Kimberly, Jeffrey C. Edberg, Donna K. Arnett, Richard A. Kaslow, Jianming Tang, Sadeep Shrestha

**Affiliations:** 1 Department of Epidemiology, University of Alabama at Birmingham, Birmingham, Alabama, United States of America; 2 Scripps Translational Science Institute, La Jolla, California, United States of America; 3 Department of Biostatistics, University of Alabama at Birmingham, Birmingham, Alabama, United States of America; 4 Clinical Immunology and Rheumatology, Department of Medicine, University of Alabama at Birmingham, Birmingham, Alabama, United States of America; 5 Department of Medicine, University of Alabama at Birmingham, Birmingham, Alabama, United States of America; The Ohio State University, United States of America

## Abstract

**Background:**

Anthrax and its etiologic agent remain a biological threat. Anthrax vaccine is highly effective, but vaccine-induced IgG antibody responses vary widely following required doses of vaccinations. Such variation can be related to genetic factors, especially genomic copy number variants (CNVs) that are known to be enriched among genes with immunologic function. We have tested this hypothesis in two study populations from a clinical trial of anthrax vaccination.

**Methods:**

We performed CNV-based genome-wide association analyses separately on 794 European Americans and 200 African-Americans. Antibodies to protective antigen were measured at week 8 (early response) and week 30 (peak response) using an enzyme-linked immunosorbent assay. We used DNA microarray data (Affymetrix 6.0) and two CNV detection algorithms, hidden markov model (PennCNV) and circular binary segmentation (GeneSpring) to determine CNVs in all individuals. Multivariable regression analyses were used to identify CNV-specific associations after adjusting for relevant non-genetic covariates.

**Results:**

Within the 22 autosomal chromosomes, 2,943 non-overlapping CNV regions were detected by both algorithms. Genomic insertions containing *HLA-DRB5, DRB1* and *DQA1/DRA* genes in the major histocompatibility complex (MHC) region (chromosome 6p21.3) were moderately associated with elevated early antibody response (β = 0.14, p = 1.78×10^−3^) among European Americans, and the strongest association was observed between peak antibody response and a segmental insertion on chromosome 1, containing *NBPF4, NBPF5, STXMP3, CLCC1*, and *GPSM2* genes (β = 1.66, p = 6.06×10^−5^). For African-Americans, segmental deletions spanning *PRR20, PCDH17* and *PCH68* genes on chromosome 13 were associated with elevated early antibody production (β = 0.18, p = 4.47×10^−5^). Population-specific findings aside, one genomic insertion on chromosome 17 (containing *NSF, ARL17* and *LRRC37A* genes) was associated with elevated peak antibody response in both populations.

**Conclusion:**

Multiple CNV regions, including the one consisting of MHC genes that is consistent with earlier research, can be important to humoral immune responses to anthrax vaccine adsorbed.

## Introduction

Anthrax, caused by *Bacillus anthracis*, is one of the most likely biological threat candidates as infection can involve the skin, gastrointestinal tract, or lungs [1], [2], [3]. Anthrax can be and has been used in biological warfare and bioterrorism. Throughout history, anthrax infections have killed hundreds of thousands of animals and people. The development of an animal vaccine in 1881 substantially reduced the threat of anthrax to livestock and humans [4]. However, anthrax still has a fatality rate of 80% or higher [5] and the long-lived, soil-borne anthrax bacterial spore remains a public health concern with intermittent outbreaks in livestock and humans. European investigators recently confirmed 14 deaths among 119 suspected anthrax cases [6]. In the United States in 2001, 22 cases of anthrax were linked to anthrax contaminated envelopes mailed to persons working in the news media and government [2]. As recently as August 2012, the Colorado Department of Agriculture investigators confirmed anthrax in one deceased cow and suspected exposure to anthrax in about 50 other dead cattle [7]. Prevention of the disease depends upon the efficacy of the licensed anthrax vaccine adsorbed (AVA-Biothrax™, Bioport Corporation, Lansing MI) [8]. AVA-Biothrax™ is a cell-free filtrate prepared from an avirulent strain of *B. anthracis,* the product contains protective antigen as its major component. It was licensed for use in 1970 after a single trial demonstrated a vaccine efficacy of 93% [9]. Like others, anthrax vaccine confers immunity by simulating a natural infection, but responses to this and other vaccines vary in human populations.

Genetic differences in vaccine response have been explained by the polymorphic nature of gene families involved in various pathways, specifically immune response. Genetic variations such as single nucleotide polymorphisms (SNPs), insertion/deletion, gene duplication and copy number variants (CNVs) are common in the human genome. Many polymorphic genetic variations have been implicated as independent cofactors in infection and immunity [10], [11], [12]. Studies among monozygotic and dizygotic twin pairs (for hepatitis B, oral polio, tetanus, *Haemophilus influenzae* type b and diphtheria vaccine response) have suggested significant heritability (44–77%) with both HLA and non-HLA genes [13], [14], [15], [16], [17]. We have previously shown that several genes, including those in the major histocompatibility (MHC) region are associated with longitudinal variation of antibody response [18], [19].

Most genetic epidemiological studies, including our own previous work on vaccine response [18], [19], have focused on SNPs. This concentration has overshadowed the importance of structural variants, especially CNVs [20]. CNVs are the result of duplications, deletions, insertions and other complex rearrangements of DNA segments often defined to be larger than 10 kb. These structural genetic variations have been shown to be involved in pathogenesis and treatment of immune related diseases [21], [22], [23], [24]. CNVs are frequently enriched in genes associated with immunity, inflammation, and host defense; they are likely under positive selection for their contribution to the enhanced ability of humans to adapt to their environment [25], [26]. We hypothesized that common CNVs play a role in differential antibody response to the AVA-Biothrax™ vaccine.

## Methods

### Ethics Statement

The parent study and this sub-study conformed to the procedures for informed written consent (parental permission was obtained wherever required) approved by institutional review boards (IRB) at all sponsoring organizations and to human-experimentation guidelines set forth by the United States Department of Health and Human Services and finally reviewed and approved by the UAB IRB.

### Study Population

The Anthrax Vaccine Research Program clinical trial (clinicaltrials.gov identifier NCT00119067, hereafter referred to as AVA000) was a multicenter, randomized, double blind trial of 1,563 healthy individuals aged 16 to 61 years at baseline and enrolled into the study between 2002 and 2008. The design of the study and participant characteristics have been described in detail previously [18], [19], [27]. Briefly, of the 1,563 participants, 1,303 were randomly assigned to seven arms: group 1 received the licensed regimen (8 doses, subcutaneously (SQ)), while group 2 also received 8 doses but with intramuscular (IM) administration. Groups 3 through 5 received between 4 and 7 IM doses, and groups 6a (SQ) and 6b (IM) received saline placebo. Of the 1,303 participants, 1,078 eligible individuals were genotyped using the Affymetrix SNP Array 6.0. All genetic and clinical data have been deposited to the Immunology Database and Analysis Portal (ImmPort) system. Based on previously reported principal component analysis [18], we selected a sample of 794 European Americans and 200 African Americans to be included in genome-wide CNV analyses.

### Measurement of IgG Antibody to Protective Antigen

IgG antibodies to protective antigen (AbPA) were measured using a quantitative enzyme-linked immunosorbent assay (ELISA) as described by Semenova et al [28]. Following administration of 3 or 4 doses of anthrax vaccine either IM or SQ, AbPA was measured at 4, 8, 26 and 30 weeks post-baseline. The impact of route of administration and number of vaccinations on the AbPA response has been discussed previously [1]. We excluded AbPA measurements taken after missed vaccinations or at times that deviated significantly from those specified in the study protocol, as previously described [18].

### Genotyping and Quality Control

Genetic data were obtained from all participating vaccinees using the Affymetrix Genome-Wide Human SNP Array 6.0. SNP genotyping methods, quality control, and association methods have been previously described in detail [19]. In the present study, we only considered interrogated CNVs. The array consisted of 744,000 probes, evenly spaced along the genome along with additional 202,000 probes targeting 5,677 CNV regions from the Toronto Database of Genomic Variants.

### CNV Calling

CNV calling algorithms can often generate varying results according to array normalization, definition of the reference genome, type of calling algorithm implemented, and parameters provided to the algorithm [29]. In this study, CNVs were called using two algorithms: the hidden Markov model calling algorithm PennCNV [30]; and the change-point calling algorithm GeneSpring GX 11 [31]. To reduce false positives, we selected CNVs that were concordant between both algorithms (see below). All the samples in each batch (96 samples) were used to create a reference signal at a given SNP for CNV calls in both algorithms. All annotated CNV genomic regions were based on the hg18 (NCBI) human genome assembly.

### PennCNV Software and Algorithm

The PennCNV algorithm considers the total signal intensity and allelic intensity ratio at each SNP, the distances between SNPs, and the frequency of each SNP via a Hidden Markov model (HMM) [30]. The PennCNV-Affymetrix protocol, as described in the manufacturer’s guide, (http://www.openbioinformatics.org/penncnv/penncnv_tutorial_affy_gw6.html) was utilized for transforming the intensities from the raw CEL files into log R ratios (LRR) and B allele frequencies (BAFs). The software’s default settings for the HMM were employed.

### GeneSpring Software and Algorithm

GeneSpring software [31] utilizes a Circular Binary Segmentation (CBS) algorithm to smooth the outliers and define change points (using default p-value <0.002 for a t-test) to identify segment breaks [32]. Once the segment breaks are identified, CNVs and confidence scores are assigned.

### Concordance of Detected CNV

PennCNV produces a discrete, integer copy number state of 0, 1, 2, 3 or 4 copies. Generally, 0 represents deletion in both chromosomes; 1, deletion in one chromosome; 3, insertion in one chromosome; and 4, two or more insertions. GeneSpring provides a continuous value that does not always clearly discriminate between single and double deletions or insertions. In order to assess concordance between the two algorithms, we classified the calls from both algorithms as either deletion, insertion, or normal without accounting for single or double insertion/deletion.

### CNV and CNV Region

We determined CNV regions (CNVRs) using the method suggested in PLINK [33]: we defined CNV predictor variables at individual genomic positions when an individual had a CNV that spanned the given position. The genomic positions used were the transition points, that is, all starting and ending positions of the called CNV for any individual in the sample. We only considered CNVs with at least two consecutive CNV probes spanning at least 10 bp showing consistent deletion or duplication. We further limited the analysis to transition points that were spanned by CNVs called in at least 10 individuals. When a test of association at a transition point was of interest (statistically significant), we defined the spanning CNVR as the largest common region containing all CNVs that spanned the given transition point.

### Statistical Analysis

The outcome variable analyzed for association with each CNV was base 10 logarithm (log_10_) AbPA at 8 weeks for early antibody response and at 30 weeks for peak antibody response. Values below detection threshold were treated as missing and excluded from the analysis. We regressed outcomes on the transition point of each confirmed CNV in our analyses. The CNV data or each transition point defined above were represented in the linear regression as either: a) any deletion versus all others (D2-); or b) any insertion versus all others (I2+). All analyses in each CNV transition point were performed separately among European American and African American populations. Adjustment for age, sex, route of administration (SQ vs. 3-IM vs. 4-IM), and the first three principal component values, from the combined population, were applied in all models [19]. For significantly associated CNV transition points, all genes were identified within the respective CNVR. As described above, administrations were performed by either 3 or 4 doses of anthrax vaccine by IM or SQ; thus we also performed separate analysis for each vaccine delivery group and performed a meta-analysis among European Americans and African Americans separately, using the DerSimonian and Laird’s linear random-effects model with the “rma” function in R package “metaphor” [34], [35]. Since CNV were relatively less frequent, when we performed the analysis in the three vaccine administration groups separately, we were under-powered to observe the associations in each group; however, we estimated the beta and p-values from the meta-analysis and confirmed the direction of the association, both in separate and overall meta-analysis to the main analysis with adjustment for the route of vaccine administration.

## Results

**Figure 1:**
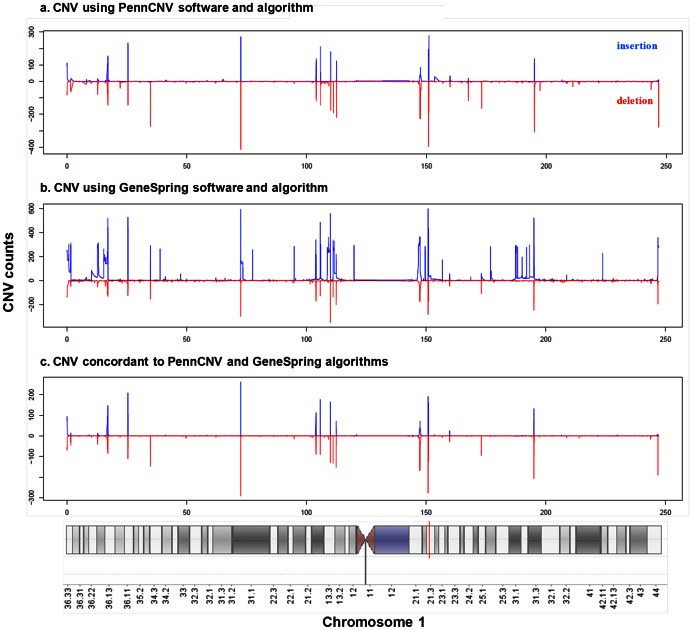
Distribution of insertion and deletion CNVs across chromosome 1 among European Americans: a) CNV calls based on the hidden Markov model calling algorithm PennCNV, b) CNV calls based on the change-point calling algorithm GeneSpring GX 11, and c) CNV calls based on the concordance between PennCNV and GeneSpring. On the vertical axis, the top peaks (blue) demonstrate the actual count of insertions and the bottom peaks (red) demonstrate the actual count of deletions. The X axis shows chromosome 1 location.

The characteristics of the 794 European American and 200 African American participants included in the analysis have been described in detail previously [18]. There were 2,943 concordant, non-overlapping, unique CNVRs spanning at least 10 bp and with a minimum count of 10 individuals in the study population that were used in the analyses (Figure 1).

**Figure 2:**
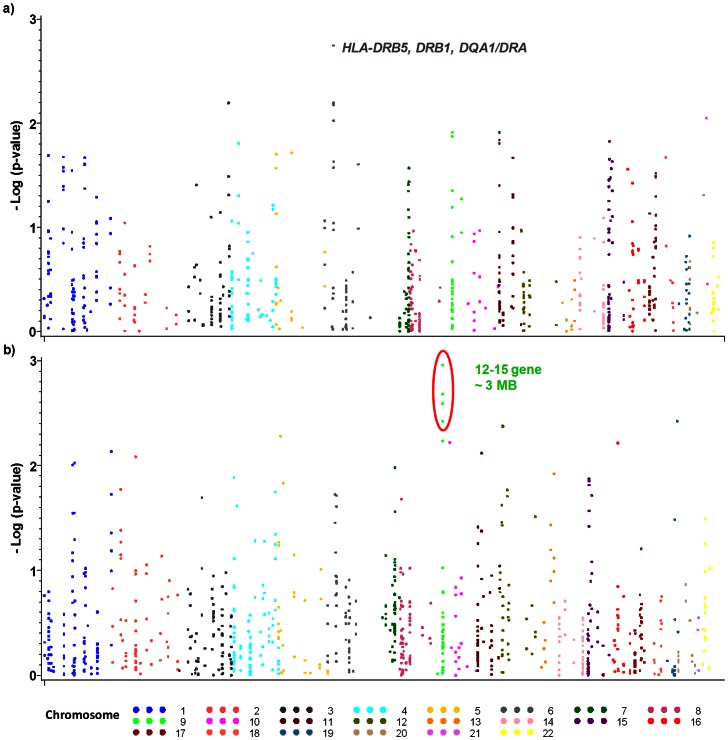
Genome-wide CNV association for early anthrax antibody response among European Americans: a) any insertions at 8 week AbPA titer, b) any deletions at 8 weeks AbPA titer. Association was assessed using linear regression models adjusted for age, sex, route of administration (SQ vs. IM), and the principal components analysis (PCA) of ancestry. The X axis shows chromosomal locations, and P values were plotted on the Y axis using logarithmic scale.

**Figure 3:**
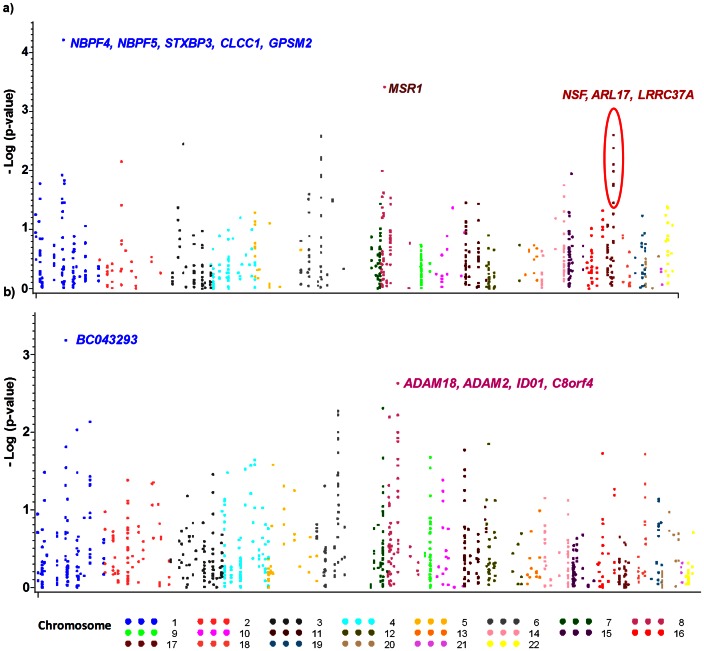
Genome-wide CNV association for peak anthrax antibody response among European Americans: a) any insertions at 30 week AbPA titer, and b) any deletions at 30 week AbPA titer. Association was assessed using single linear regression adjusted for age, sex, route of administration (SQ vs. IM), and the principal components analysis (PCA) of ancestry. The X axis shows chromosomal locations, and P values were plotted on the Y axis using a logarithmic scale.

The genome-wide association signals for concordant insertion and deletion CNVs with antibody response among European Americans for early vaccine response at week 8 are shown in Figures 2a and 2b, respectively; and at week 30 for peak vaccine response in Figures 3a and 3b, respectively (all results presented in table S1). The strongest significant associations of CNVs (confirmed by both adjusted analysis and meta-analysis) with early and peak anthrax antibody responses are displayed in Table 1. Among European Americans, the genomic insertions in the positions – chr6∶32,539,530–32,701,486 (Figure 2a) are associated with early elevated antibody response (β = 0.14; p = 1.78×10^−3^). This CNVR contains *HLA-DRB5, DRB1, DQA1/DRA* genes. Similarly, insertions of genomic regions chr8∶16,251,325–16,333,182, and chr1∶108,729,983–109,312,876 (Figure 3a) have moderate effect on elevated peak antibody (β = 0.61; p = 9.29×10^−3^ and β = 1.66; p = 6.06×10^−5^ respectively). The chromosome 1 region contains multiple genes - *NBPF4, NBPF5, STXBP3, CLCC1, and GPSM2*. Also, deletions in chr1∶105,814,277–106,015,829, containing *BC043293* gene as well as deletions in the CNVR chr8∶39,344,897–40,302,956 (Figure 3b), containing *ADAM18, ADAM2, ID01, C8orf4* genes are associated with elevated peak antibody response.

**Table 1:** CNV regions associated with week 8 and week 30 IgG antibody response to anthrax vaccine adsorbed in European Americans and African Americans.

	CNVtype	Chr	CNV Marker point*		CNV probe ID†		Common CNV genomic region*	Gene	Adjusted analysis Beta p		Meta-analysis Beta p	
**European American**												
**Early response** **(8 weeks)**												
	I2+	6	*32,562,253*			*CN_1175484*	32,539,530–32,701,485	*HLA-DRB5, DRB1, DQA1/DRA*	0.14	1.78E-03	0.15	0.01
	D2−	9	43,542,722			CN_1322511	41,682,297–44,780,914	12–15 loci	−0.12	1.10E-03	−0.15	0.05
**Peak response** **(30 weeks)**												
	D2−	1	105,826,321			SNP_A-8310560	105,814,277 -106,015,829	*BC043293*	1.04	6.52E-04	1.33	1.87E-04
	I2+	1	*109,298,377*			*CN_451797*	108,729,983–109,312,876	*NBPF4, NBPF5, STXBP3, CLCC1, GPSM2*	1.66	6.06E-05	1.70	5.28E-05
	D2−	8	*39,452,128*			*CN_1283719*	39,344,897–40,302,956	*ADAM18, ADAM2, ID01, C8orf4*	0.12	2.35E-03	0.13	1.97E-03
	I2+	17	*42,078,158*			*CN_739340*	41,521,621–42,150,374	*NSF, ARL17, LRRC37A*	0.12	2.48E-03	0.13	1.02E-03
**African American**												
**Early response** **(8 weeks)**												
	D2−	13	56,661,148	CN_654225			54,593,069–58,010,252	*PRR20, PCDH17, PCH68*	0.18	4.47E-05	0.21	8.57E-04
	D2−	13	68,146,300	SNP_A-8363651			68,140,180–68,166,243	*PCDH9, KLHL1*	−0.82	1.79E-03	−0.81	1.47E-03
**Peak response** **(30 weeks)**												
	I2+	3	*164,106,591*	*CN_991857*			163,985,816–164,117,009	*BC073807*	0.29	1.25E-03	0.51	2.49E-03
	I2+	5	*886,628*	*CN_1131184*			751,371–1,048,270	*ZDHHC11, BRD9, TRIP13*	−0.27	2.73E-03	−0.22	3.23E-03
	D2−	11	*55,133,210*	*CN_587565*			55,108,112–55,414,942	*OR4C11, OR4P4, SPRYD5*	−0.33	1.46E-03	−0.36	2.76E-03
	I2+	17	*42,120,174*	*CN_739357*			41,521,621–42,150,374	*NSF, ARL17, LRRC37A*	0.60	2.14E-04	0.11	2.89E-03

I2+ = any insertion, D2- = any deletion;

* genomic position based on hg18;

† CNV probe ID based on the Affymetrix 6.0 microarray.

With the smaller sample-size of African American populations in our study, all results are presented in table S2. Of special interest in African Americans, deletions in the CNVR chr13∶54,593,069–58,010,252 containing *PRR20, PCDH17, PCH68* genes (Table 1 and Table S2) are significantly associated with increased antibody levels in the early period (Table 1 - β = 0.18, p = 4.47×10^−5^). In contrast, deletions in the CNVR chr13∶68,140,180–68,166,243 that contains *PCDH9, KLHL1* genes are associated with reduced early antibody response. For the peak response, insertions in the genomic region chr3∶163,985,816–164,117.009 that contains *BC073807* gene (Table 1 and Table S2) are associated with mildly increased antibody response (β = 0.24; p = 1.25×10^−3^). Interestingly, in both European Americans and African Americans, insertions in the genomic region chr17∶41,521,621–42,150,374 (Table 1) are associated with elevated peak antibody response. This region consists of *NSF, ARL17 and LRRC37A* genes.

## Discussion

Vaccination against anthrax is very effective with generally high but variable antibody response. Using genome-wide association testing, we focused on CNV, an important but not yet commonly evaluated source of genetic variation, for its association with AbPA response to AVA-Biothrax™ in European American and African Americans. The strongest associations were found in regions containing *NBPF4, NBPF5, STXBP3, CLCC1, GPSM2* on chromosome 1 (adjusted analysis p = 6.06×10^−5^, meta-analysis p = 5.28×10^−5^) in European Americans, and in regions containing *PRR20, PCDH17, PCH68* (adjusted analysis p = 4.47×10^−5^, meta-analysis p = 8.57×10^−4^) in African-Americans. CNVs containing MHC class II genes, namely *HLA–DRB5, DRB1, and DQA1/DRA* on chromosome 6 were also identified on chromosome 6 in European Americans only. Interestingly, CNVs containing *NSF, ARL17 and LRRC37A* genes on chromosome 17 were associated with elevated antibody response in subjects of both races.

The association of MHC class II genes -*HLA–DRB1, DQA1* with anthrax vaccine antibody response has been previously reported in SNP analysis [18]. In a GWAS of hepatitis B vaccine response, markers in *HLA-DR* and *HLA-DP* showed independent associations with antibody response [36]. Similarly, markers in *HLA-DRB5* were found to be associated with elevated antibody response. In our own previous study in this population [37], carriage of three haplotypes in this region (DRB1*1501-DQA1*0102-DQB1*0602, DRB1*0101-DQA1*0101-DQB1*0501, and DRB1*0102-DQA1*0101-DQB1*0501) were all significantly associated with lower AbPA levels in the longitudinal analyses [18]. Recently in an animal study [37], MHC class II locus has been associated with high antibody titers to a component of anthrax toxin, lethal factor whereas non-MHC class loci were discovered to influence antibody titers to protective antigen.

Interestingly, as an internal replication, CNVs in the genomic region for chromosome 17 containing *NSF, ARL17, LRRC37A* genes were associated with increased antibody response in both European Americans and African Americans. The *NSF* gene, N-ethylmaleimide-sensitive factor, is one of the essential components of the cellular membrane fusion machinery, an important homeostatic process [38]. NSF gene has been associated with Parkinson’s disease [39]. In one study *ARL17*, ADP-ribosylation factor-like 17 was one of the genes reported to be differentially regulated in the human monocytes after treatment with anthrax lethal toxin (LT) [40]. ARL17 is localized to the Golgi apparatus and is thought to be involved in modulation of vesicle budding and is known to be an activator of cholera toxin catalytic subunit, an ADP-ribosyltransferase [41]. *LRRC37A* (leucine-rich repeat containing 37A) belongs to the complex LRRC37 family that has been mapped to 18 distinct loci in the human genome on the q arm of chromosome 17. LRRC37A protein localizes to cellular vesicles, including Golgi apparatus and to the plasma membrane [42]. Variants in *LRC37A* have also been associated with Parkinson disease [43]. However, to date there are no reports of involvement of any of these genes in immunity or vaccine response.

We also observed associations with other CNVRs (table 1); but none of the genes within those CNVRs have a known role in basic immunology or vaccine response mechanisms. *STXBP3* and *CLCC1* genes on chromosome 1 have been linked to insulin dependent diabetes mellitus [44]. *STXBP3*, syntaxin binding protein, is associated with glucose metabolism in adipocytes and contributes to fusion of intracellular GLUT4-containing vesicles with the cell surface in adipocytes [44]. *CLCC1*, chloride channel CLIC-like 1, produces protein located in the membranes of intracellular compartments of endoplasmic reticulum and Golgi apparatus [45]. *GPSM2*, G-protein-signaling modulator 2, belongs to a family of genes that modulate G proteins activation and is associated with cell division and cancer [46]. *NBPF4* and *NBPF5* are neuroblastoma breakpoint family genes that have not been associated with any disease.

Each CNV calling algorithm and analytical approach has its own limitations and strengths. In our study, Penn-CNV identified 5,812 different CNVRs, with an average of 70 CNVs in each individual; whereas GeneSpring identified 7,271 different CNVRs, with an average of 168 in each individual. Using only the CNVs with concordant calls from two different methods will miss potential CNVs from the filtering due to inherent difficulties in defining CNVs. For example, the HMM algorithm has difficulty with heterogenous CNV regions. However, the requirement for concordance provides higher confidence in the fidelity of the CNV calls. CNVs consistently called from two or more different calling algorithms are more reliable than those CNVs called from a single algorithm [47]. Even with our more stringent definition, the number of CNV calls per person seems to be higher in our study than other studies, very likely because we have considered CNVs as small as 10 bp (but occurring in at least 10 individuals), rather than the conventional size of 10 kb that is often used. However, that difference is not a major concern for our analysis because most of the significant associations were observed with large CNVs.

Of note, in this study we examined the association of the CNVs in both early and peak vaccine response. The biological mechanisms of early and peak response may well be different, and our studies suggest that CNVs associated with differential levels of antibody were different at the two phases. Our study involved two ethnic populations nested within a multi-center vaccine trial. While this cohort is the largest for an anthrax vaccine genetic study, our analysis in African Americans remains underpowered. Nevertheless, one region of the genome seems to have a constant effect in both European American and African American populations. With our conservative approach to CNV calls, we only examined 2,943 CNVs in total; thus, our associations seem to meet the stringent correction criteria for multiple tests. As with most vaccine genetic studies, another limitation of the present study is lack of a readily available replication cohort. Although the results from this study are preliminary, they show the potential for identification of gene copy number that could play a role in vaccine response. Weaponized anthrax spores remain a lethal bioterrorism threat to military personnel and civilian populations; continued research is therefore needed to understand whether and how genetic profiles can predict vaccine response.

## Supporting Information

Table S1 **Complete association results of all CNV tranisition points with early (8 weeks) and peak (30 weeks) antibody responses to anthrax vaccine in 794 European Americans.**
(XLSX)

Table S2 **Complete association results of all CNV tranisition points with early (8 weeks) and peak (30 weeks) antibody responses to anthrax vaccine in 200 African Americans.**
(XLSX)
